# Alkali-metal-adsorbed g-GaN monolayer: ultralow work functions and optical properties

**DOI:** 10.1186/s11671-018-2625-z

**Published:** 2018-07-11

**Authors:** Zhen Cui, Xia Wang, Enling Li, Yingchun Ding, Changlong Sun, Minglei Sun

**Affiliations:** 1 School of Automation and Information Engineering, Xi’an University of Technology, Xi’an, 710048 People’s Republic of China; 2 College of Optoelectronics Technology, Chengdu University of Information Technology, Chengdu, 610225 People’s Republic of China; 3 Department of Radiology, Affiliated Hospital of Yan’an University, Yan’an, 716000 People’s Republic of China; 4 State Key Lab of Crystal Materials, Shandong University, Jinan, 250100 Shandong People’s Republic of China; 5 School of Mechanical Engineering, Southeast University, Nanjing, Jiangsu 211189 People’s Republic of China; 6 Institute of High Performance Computing, A*STAR, Singapore, 138632 Singapore

**Keywords:** G-GaN, Adsorption, Work function, Field emission device, Optical properties, Density functional theory

## Abstract

The electronic and optical properties of alkali-metal-adsorbed graphene-like gallium nitride (g-GaN) have been investigated using density functional theory. The results denote that alkali-metal-adsorbed g-GaN systems are stable compounds, with the most stable adsorption site being the center of the hexagonal ring. In addition, because of charge transfer from the alkali-metal atom to the host, the g-GaN layer shows clear n-type doping behavior. The adsorption of alkali metal atoms on g-GaN occurs via chemisorption. More importantly, the work function of g-GaN is substantially reduced following the adsorption of alkali-metal atoms. Specifically, the Cs-adsorbed g-GaN system shows an ultralow work function of 0.84 eV, which has great potential application in field-emission devices. In addition, the alkali-metal adsorption can lead to an increase in the static dielectric constant and extend the absorption spectrum of g-GaN.

## Background

Compared with traditional semiconductor materials, three-dimensional GaN is a wide-bandgap semiconductor material [1]. As such, it can enable equipment operation at ultra-high voltage, frequency, or temperature and exhibits high luminous efficiency, good thermal conductivity, high temperature resistance, resistance to acids and alkalis, and anti-radiation properties. As an optoelectronic material, three-dimensional GaN has potential applications in laser printing and high-storage-density compact discs, potentially strongly influencing the technology of computer storage [2]. In recent years, two-dimensional (2D) materials have received extensive attention because of their fascinating optical, mechanical, electronic, and magnetic properties and potential for multifunctional applications [3–9]. 2D materials are far thinner than bulk materials, and the mechanical, electronic, thermal, and optical properties of such materials differ substantially from those of their bulk counterparts [10]. Specifically, 2D GaN is a wide-bandgap material with enhanced optoelectronic performance. Very recently, it was synthesized via a migration-enhanced encapsulated growth technique [11].

Studying and understanding the interaction between atoms on solid surfaces is one of the basic scientific problems in the field of surface physics. Therefore, controlling such self-assembling structures is important for the development of nanodevices. Atoms adsorbed onto a solid surface can interact indirectly through electron scattering or elastic distortion of the substrate, with the long-range atomic interaction modulated by the substrate playing an important role in atomic self-assembly. Because alkali-metal atoms can easily lose electrons, the adsorption of alkali metals onto semiconductor materials can change them to n-type, which will in turn reduce their work function and change their optoelectronic properties [12]. In recent years, many research groups have reported studies of the optoelectronic properties of alkali-metal-adsorbed 2D materials [13–23]. For instance, Chan et al. [13] investigated the adsorption of alkali-metal atoms on graphene and discovered the reduction of work function of graphene. Jin et al. [14] and Qiao et al. [15] investigated the adsorption of alkali metals on graphene using first-principles method and found that the optoelectronic properties of graphene are modified by the adsorption of alkali-metal. Many previous works investigated that the electronic and magnetic properties of adatom adsorptions on black and blue phosphorene, which found the surface adsorptions effectively functionalize the phosphorene system with versatile spintronic features [16–18]. However, the full photoelectric properties of alkali-metal-adsorbed g-GaN are still not clear.

In this article, the band structures, density of states, work functions, and optical properties of pristine g-GaN and alkali-metal-adsorbed g-GaN are elaborated; this research is potentially important for the fabrication of g-GaN-based field-emission and optoelectronic devices.

## Methods

All the calculations are performed by using the Vienna Ab initio Simulation Package based on first-principles with density functional theory [24]. The generalized gradient approximation (GGA) in the form of the Perdew-Burke-Ernzerhof (PBE) functional [25] was adopted to describe the exchange-correlation interaction. The GGA-PBE method has been indicated to be very effective for surface research [26–29]. The kinetic cutoff energy for the plane-wave basis set is 500 eV. In the perpendicular direction of the g-GaN plane, the vacuum space was set to 20 Å. The Brillouin zone was described by a set of *k*-points in a 9 × 9 × 1 grid using the Γ-centered scheme. All atoms are fully relaxed until the Hellmann–Feynman forces were less than10^− 4^ eV/Å and the total energy changes became less than 10^− 4^ eV [29].

The adsorption energy for the alkali-metal-adsorbed g-GaN systems was calculated using the method of Cui et al. [12] According to the following equation:

1 $$ {E}_{ads}={E}_{g- GaN\ \mathrm{X}}-{E}_{g- GaN}-{\mu}_X, $$

where *E*_ads_ is the adsorption energy, *E*_g-GaN_ and *E*_g-GaN:X_ denote the total energy of pristine g-GaN before and after alkali-metal adsorption, respectively, and μ_X_ is the chemical potential of a single alkali-metal atom. Based on this equation, a negative value of *E*_ads_ denotes a stable structure.

The charge-density difference is described as

2 $$ \varDelta \rho ={\rho}_T-{\rho}_g-{\rho}_x, $$

where *ρ*_T_, *ρ*_g_, and *ρ*_x_ are the total charge on the alkali-metal-adsorbed g-GaN, pristine g-GaN, and adsorption atom, respectively.

## Results and discussions

**Fig. 1:**
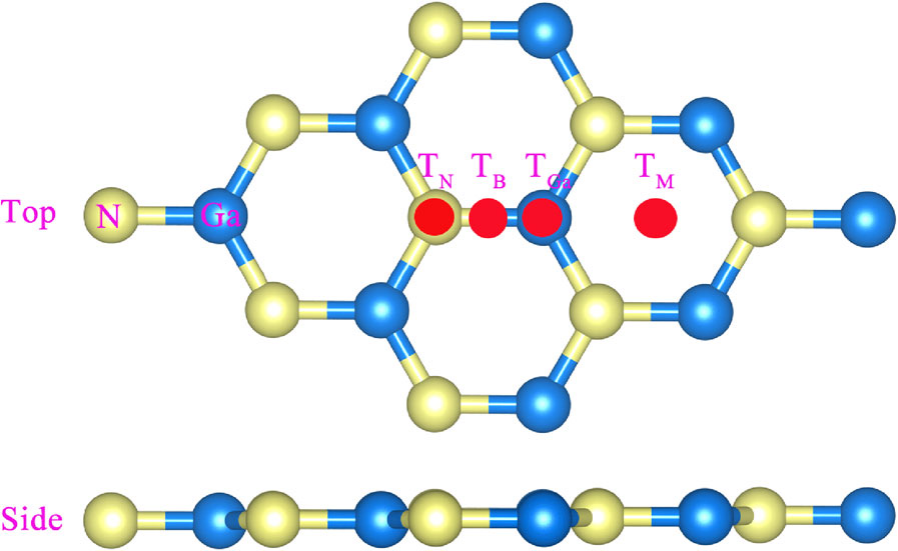
Model of g-GaN with different adsorption sites

Figure 1 shows the model of g-GaN for four different adsorption sites; the T_N_ site is directly above the N atom, the T_Ga_ site is directly above the Ga atom, the T_B_ site is above the middle of the N-Ga bond, and the T_M_ site is above the center of a hexagon. The calculated *E*_ads_ of alkali-metal-adsorbed g-GaN is shown in Table 1. All the *E*_ads_ of different sites are negative, which demonstrates that the procedure of alkali-metal adsorption on g-GaN is exothermic and all the adsorption systems are stable. These results are similar to those obtained for alkali-metal-adsorbed GaN nanowires [12]. Moreover, the calculated results indicate that the most stable position is the T_M_ site; thus, the following discussions only concern the T_M_ adsorption site.

**Table 1:** The adsorption energy for alkali-metal-adsorbed g-GaN with different sites

Adsorption style	*E*_ads_T_Ga_ (eV)	*E*_ads_T_B_ (eV)	*E*_ads_T_N_ (eV)	*E*_ads_T_M_ (eV)
Li	− 1.89	− 1.90	− 1.26	− 1.92
Na	− 1.07	− 1.12	− 0.76	− 1.18
K	− 1.07	− 1.09	− 0.81	− 1.21
Rb	− 0.94	− 0.98	− 0.77	− 1.07
Cs	− 0.94	− 1.03	− 0.71	− 1.08

The lattice parameters of pristine and alkali-metal-adsorbed g-GaN are shown in Table 2. The lattice parameters of pristine g-GaN are 3.254 Å, which are in good agreement with previous results [30–33]. Furthermore, the lattice parameters of Li- or Na-adsorbed g-GaN are little smaller than that in pristine g-GaN, whereas the K-, Rb-, and Cs-adsorbed g-GaN are bigger than that in pristine g-GaN. Interestingly, as the atomic number of alkali metal atoms tunes larger, the lattice parameters of alkali-metal-adsorbed g-GaN increase. The bond lengths of the N-X or Ga-X are displayed in Table 2. The bond lengths of the N-X or Ga-X increase with the increasing of atomic number of alkali metal atoms. The adsorption height of alkali-metal-adsorbed g-GaN are shown in Table 2, which indicate that the adsorption height increase with the increasing of atomic number of alkali metal atoms.

**Table 2:** The lattice parameters, bond length, and adsorption height of pristine and alkali-metal-adsorbed g-GaN

Adatom	Pristine	Li	Na	K	Rb	Cs
d_N-X_ (Å)	/	2.154	2.511	2.917	3.084	3.249
d_Ga-X_ (Å)	/	2.635	2.975	3.371	3.530	3.701
Lattice parameters	3.254	3.241	3.253	3.259	3.261	3.268
Adsorption height (Å)	/	2.501	2.718	3.102	3.335	3.598

**Fig. 2:**
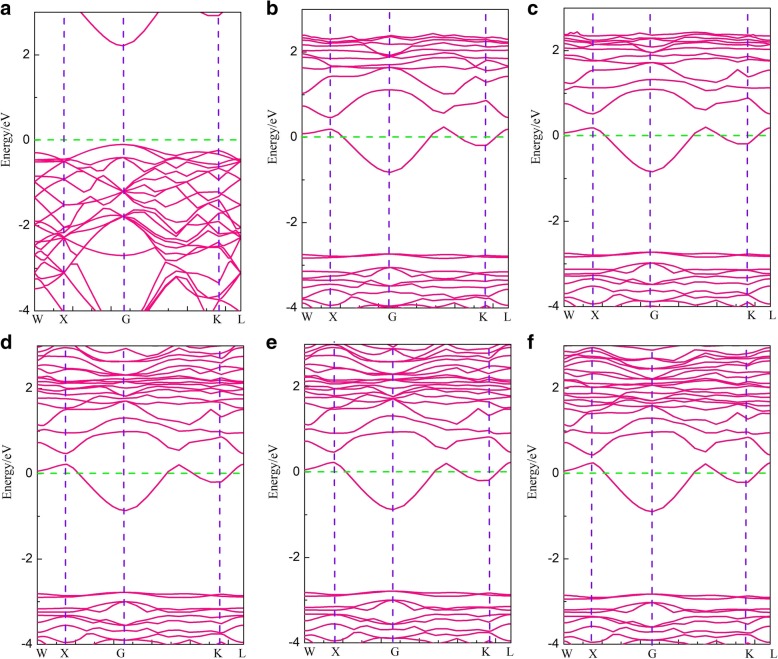
Band structures for pristine and alkali-metal-adsorbed g-GaN: **a** pristine g-GaN, **b** Li-adsorbed g-GaN, **c** Na-adsorbed g-GaN, **d** K-adsorbed g-GaN, **e** Rb-adsorbed g-GaN, and **f** Cs-adsorbed g-GaN. The Fermi level is denoted by green dashed lines

The band structures of pristine and alkali-metal-adsorbed g-GaN are shown in Fig. 2. Figure 2a clearly shows that the band structure of pristine g-GaN exhibits semiconducting character, with a bandgap of 2.1 eV. This result is in good agreement with the previous reports [30–33]. However, the band structures for alkali-metal-adsorbed g-GaN show that the Fermi levels have entered the conduction band, as shown in Fig. 2b–f; thus, the alkali-metal-adsorbed g-GaN system features a metallized character, with a gap appearing at approximately − 1.8 eV under the Fermi level, and the gap of alkali-metal-adsorbed g-GaN is approximately 1.92 eV. Furthermore, the g-GaN is transformed into an n-type semiconductor after adsorption of the alkali metals because of the tendency for the alkali metals to lose electrons, resulting in an upshift of the Fermi level inside the conduction band.

**Fig. 3:**
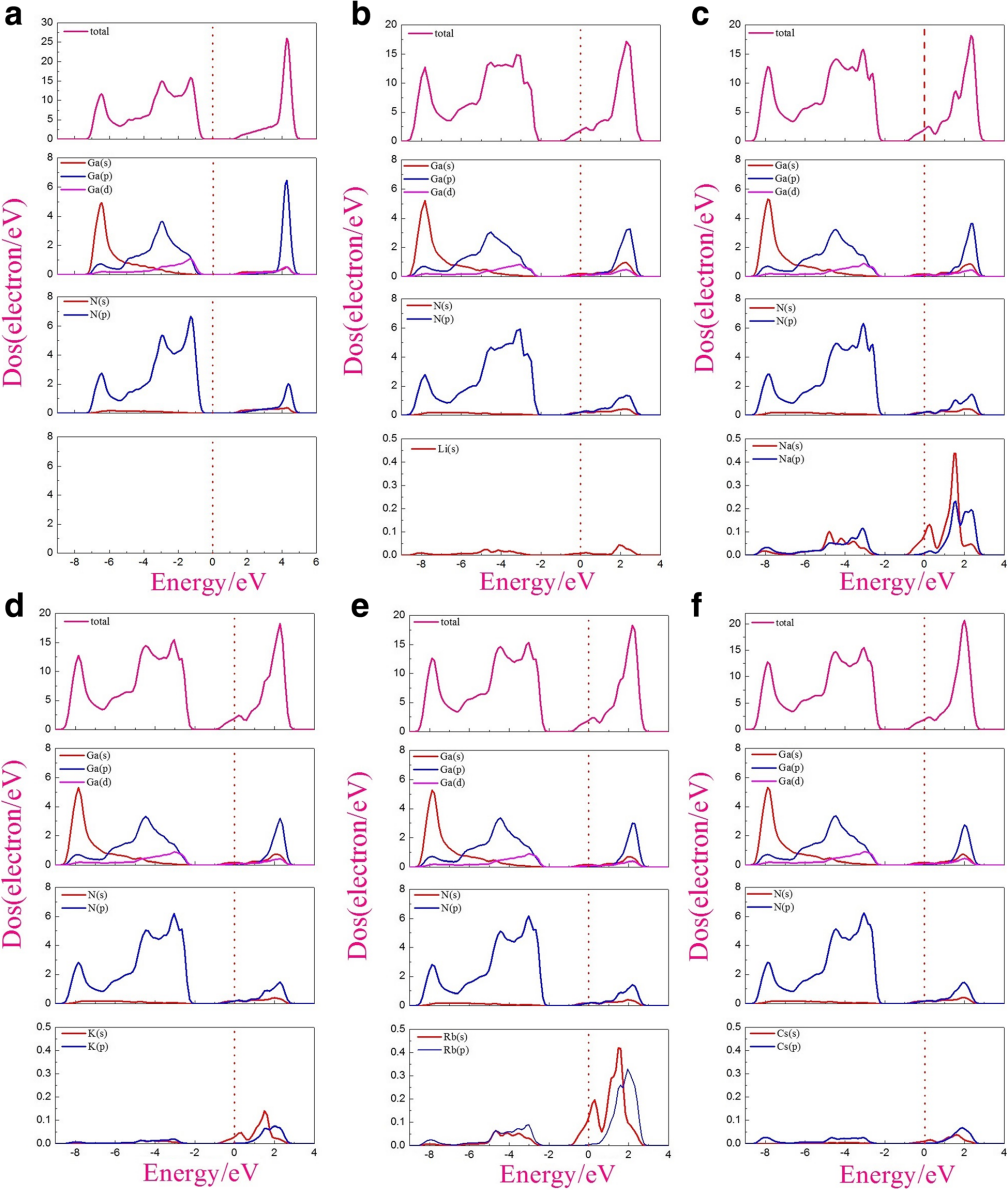
Density of states for pristine and alkali-metal-adsorbed g-GaN: **a** pristine g-GaN, **b** Li-adsorbed g-GaN, **c** Na-adsorbed g-GaN, **d** K-adsorbed g-GaN, **e** Rb-adsorbed g-GaN, and **f** Cs-adsorbed g-GaN

The total density of states (TDOS) and partial density of states (PDOS) of pristine and alkali-metal-adsorbed g-GaN are shown in Fig. 3. In Fig. 3a, the TDOS of pristine g-GaN demonstrates that it is a semiconductor, consistent with the result of band structure. The PDOS calculations show that the valence-band maximum for pristine g-GaN originates from the N-2p and Ga-4p orbitals, in agreement with the previous results [34, 35]. To understand the electron states near the Fermi level, we calculated the PDOS of alkali-metal-adsorbed g-GaN. As can be seen from Fig. 3b–f, the electron states near the Fermi level are mainly governed by the Ga-4s, N-2p, and 2s orbitals of the alkali metals.

**Fig. 4:**
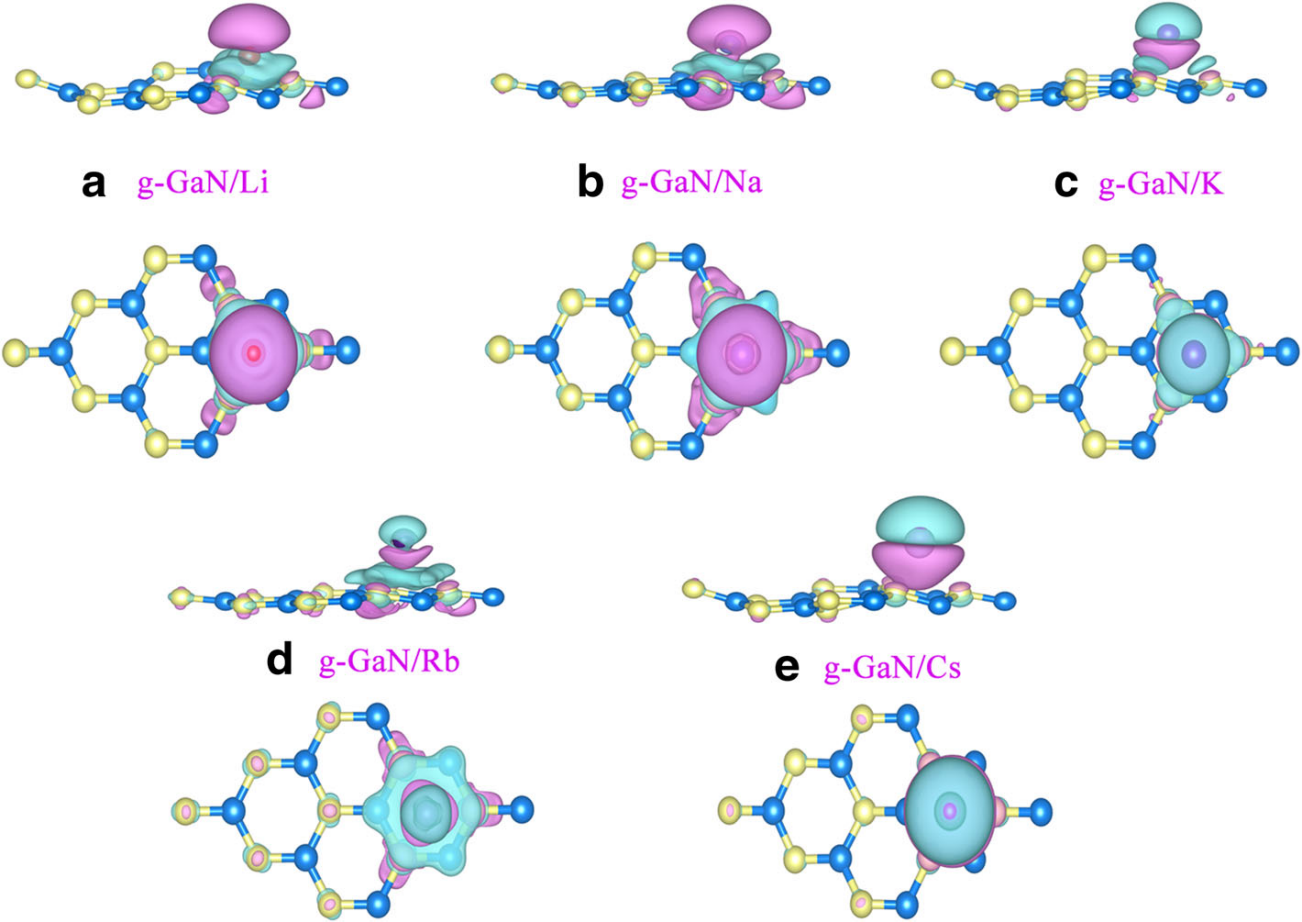
The charge-density difference for alkali-metal-adsorbed g-GaN. **a** g-GaN/Li,** b** g-GaN/Na, **c** g-GaN/K, **d **g-GaN/Rb,** e** g-GaN/Cs.The magenta and cyan regions denote the gain and loss of electrons, respectively. The value of the isosurface is set to 0.002 e/Å^3^

Charge transfer is an important aspect of the adsorption system. The charge-density difference with an isosurface value of 0.002 e/Å^3^ for alkali-metal-adsorbed g-GaN is shown in Fig. 4. Interestingly, the electron distributions lie between all the alkali-metal atoms and the three under-coordinated N atoms. Therefore, alkali-metal-adsorbed g-GaN is formed by chemisorption. In addition, the large cyan region localized on alkali-metal atom suggests a large transfer from alkali-metal atom to g-GaN. Bader charge analysis shows that there are about 0.8833|e|, 0.7803|e|, 0.7997|e|, 0.7905|e|, 0.7936|e| transfer from Li, Na, K, Rb, Cs to g-GaN. Thus, all the results above confirmed the picture that the interactions in alkali-metal-adsorbed g-GaN are ionic bonding.

**Fig. 5:**
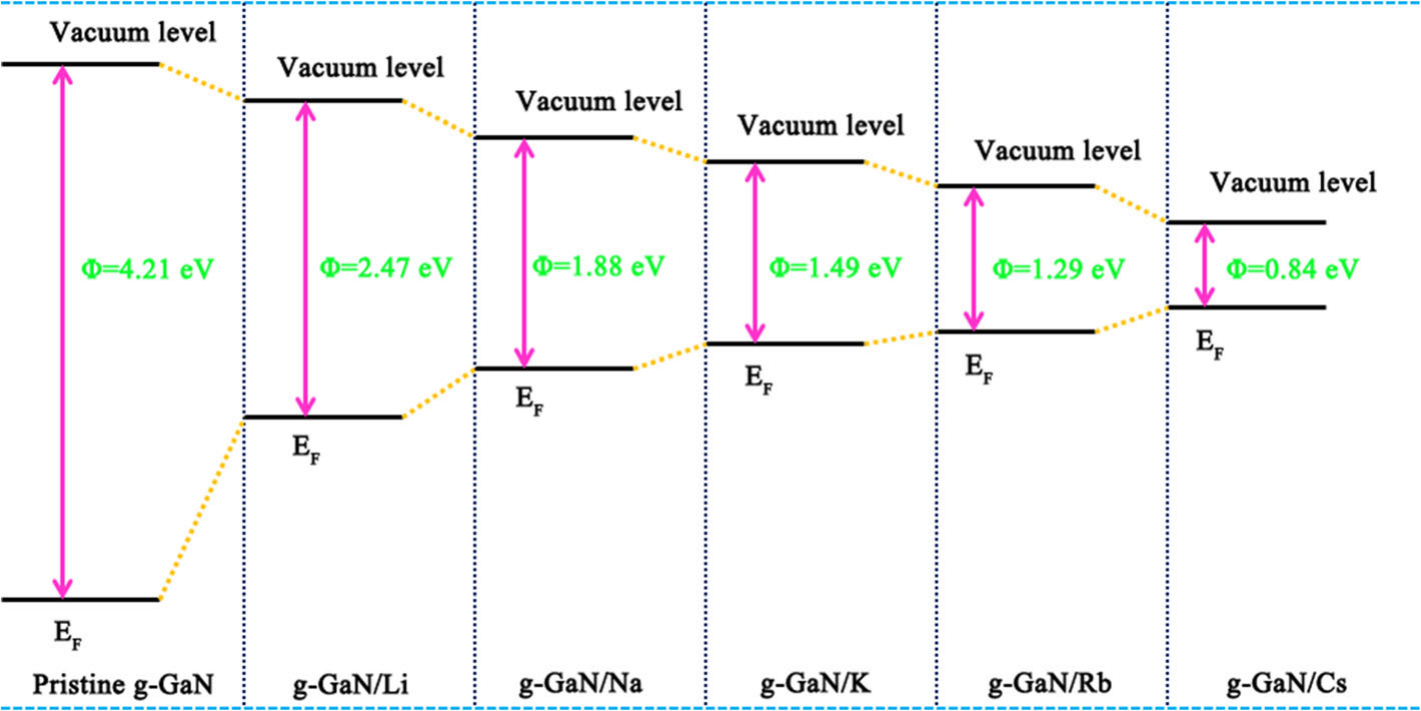
The work function schematic for pristine and alkali-metal-adsorbed g-GaN

Work function is a critical factor for balancing the optoelectronic properties of materials. The work function of materials is equal to the vacuum level deducted from the Fermi level. To reveal intriguing feasibility, we studied tuning of the work function of g-GaN by alkali-metal adsorption. Figure 5 shows the work function schematic of pristine g-GaN and alkali-metal-adsorbed g-GaN. The work function of pristine g-GaN is 4.21 eV, which is slightly larger than that of GaN nanowires [12]. The work functions are 2.47, 1.88, 1.49, 1.29, and 0.84 eV for Li-, Na-, K-, Rb-, and Cs-adsorbed g-GaN, respectively; thus, the work function of g-GaN is substantially reduced following adsorption of an alkali metal adatom. Furthermore, the work functions of alkali-metal-adsorbed g-GaN are lower than those of alkali-metal-adsorbed GaN nanowires [12]. The main reason may due to the structure difference between GaN monolayer and nanowires. Moreover, the decreased work function demonstrates that the alkali-metal-adsorbed g-GaN can be used to field-emission devices.

**Fig. 6:**
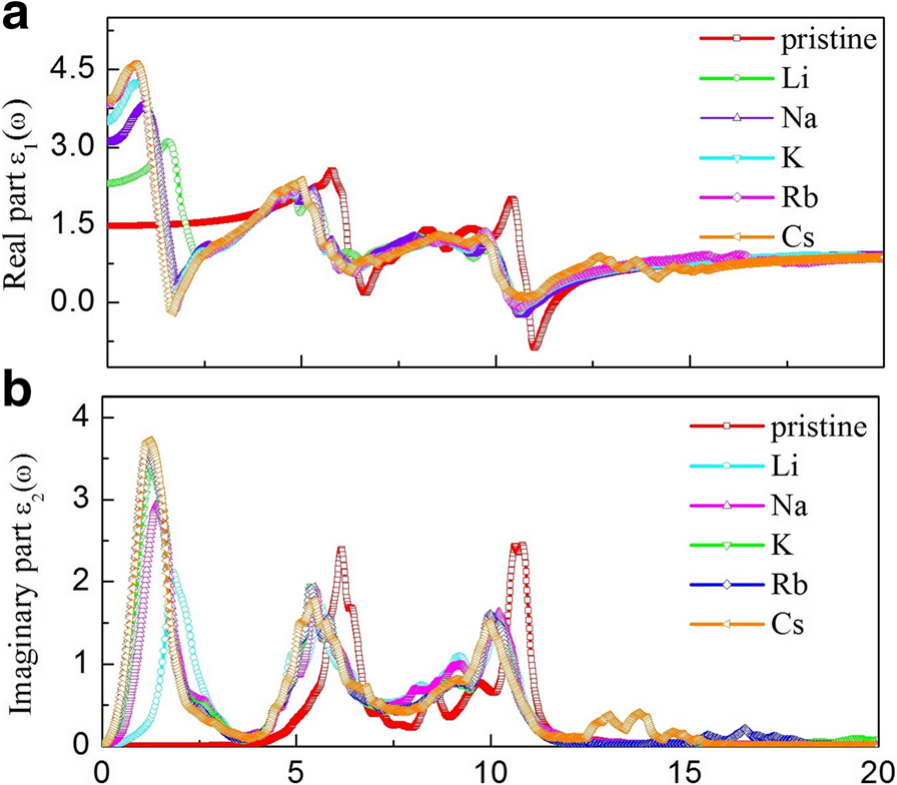
The real and imaginary parts for pristine and alkali-metal-adsorbed g-GaN:** a** real parts, **b** imaginary parts

Next, we turn to investigate the effect of the adsorption of alkali metal on the optical properties of g-GaN. Optical properties of materials can be depicted by the real part *ε*_1_(*ω*) and imaginary part *ε*_2_(*ω*) of the dielectric function, absorption *a*(*ω*), refractive *n*(*ω*), reflectivity *R*(*ω*), energy-loss function *L*(*ω*), and the extinction coefficient spectra *K*(*ω*), as reported previously [36–40]. The real part *ε*_1_(*ω*) as a function of *ω* for pristine and alkali-metal-adsorbed g-GaN is shown in Fig. 6a. The *ε*_1_(*0*) of pristine g-GaN is 1.48, and the *ε*_1_(*0*) of alkali-metal-adsorbed g-GaN is 2.33 (Li), 3.13 (Na), 3.56 (K), 3.81 (Rb), and 3.81 (Cs). The data show that the *ε*_1_(*0*) of alkali-metal-adsorbed g-GaN is greater than that of pristine g-GaN; thus, the optical properties of g-GaN are highly sensitive and tunable. In addition, when the energy is greater than 15 eV, the tendency for the real part of the spectrum is identical to that corresponding to adsorption by different alkali metals. The imaginary part *ε*_2_(*ω*) as a function of *ω* for pristine and alkali-metal-adsorbed g-GaN is shown in Fig. 6b. Two narrow peaks located at 6.18 and 10.76 eV, which originate from the transition of N-2p electrons into the s states of the cations, are shifted toward lower energies upon alkali-metal adsorption. Moreover, a high peak arises at 1.22 eV following alkali-metal adsorption.

**Fig. 7:**
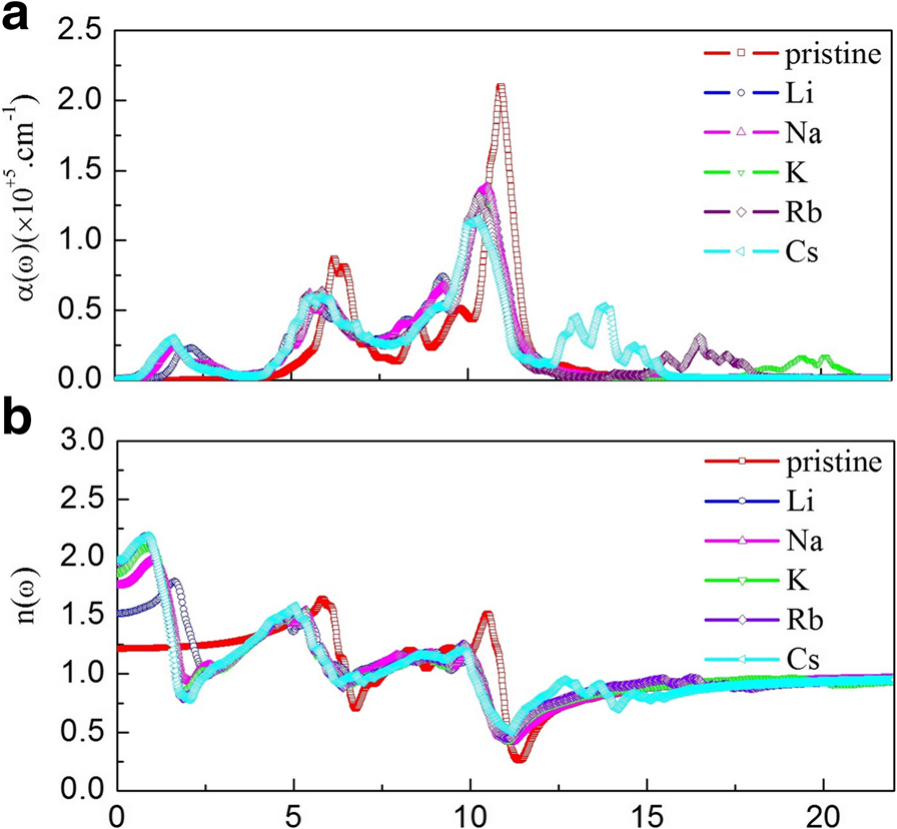
The absorption coefficient and refractive indices for pristine and alkali-metal-adsorbed g-GaN: **a** absorption coefficient, **b** refractive indices

**Fig. 8:**
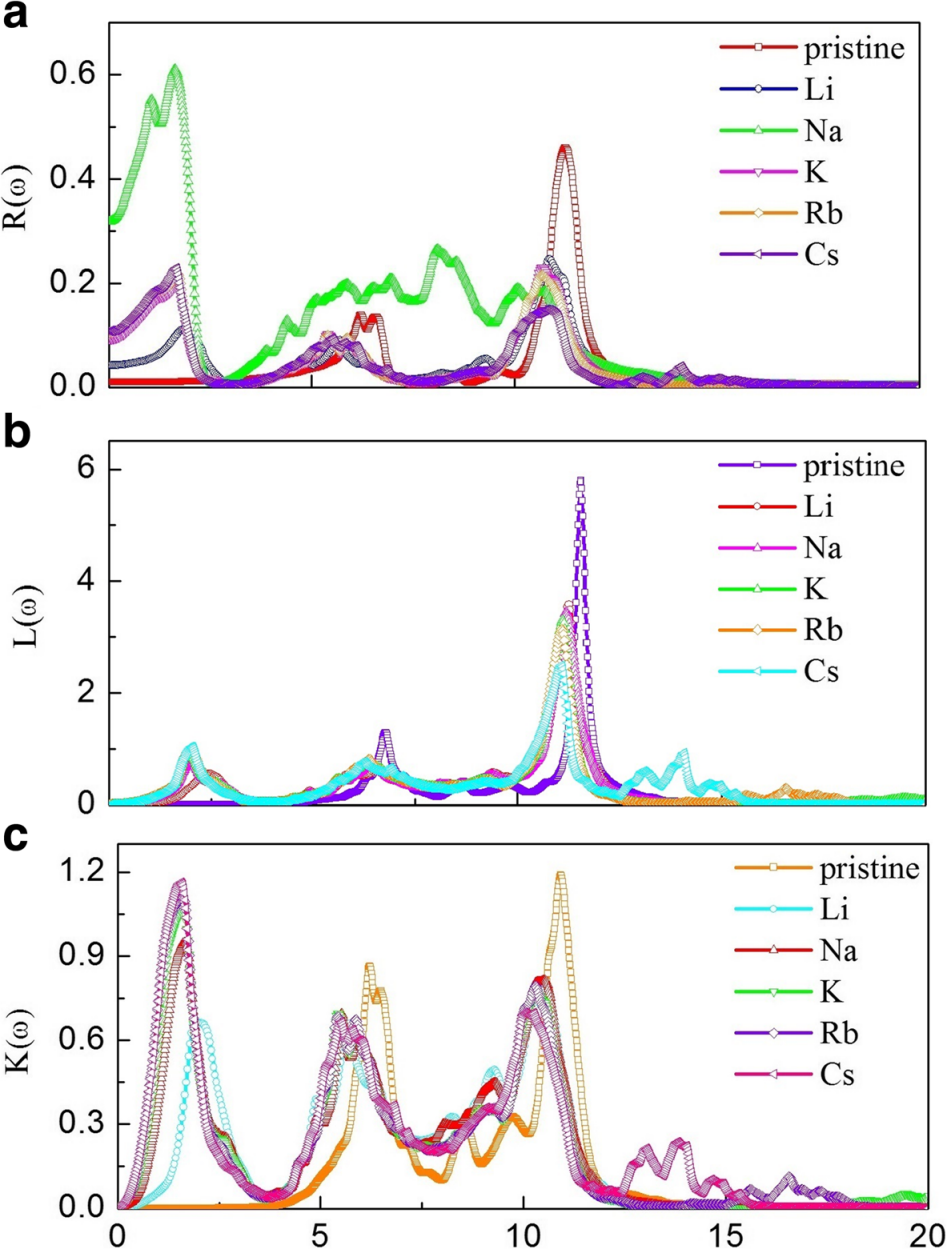
The reflectivity coefficient, loss energy function, and extinction coefficient for pristine and alkali-metal-adsorbed g-GaN: **a** reflectivity coefficient, **b**  loss energy function, **c** extinction coefficient

Figure 7 shows the absorption coefficient and refractive indices for pristine and alkali-metal-adsorbed g-GaN. In Fig. 7a, the absorption edge of pristine g-GaN begins at 2.77 eV; this absorption stems from the excited electron transition from N-2p states located at the top of the valence band to the empty cation 2 s states. The spectrum of pristine g-GaN shows two peaks located at 6.28 and 10.95 eV; these peaks exhibit a redshift after alkali-metal adsorption. In addition, the intensities the two peaks decrease following alkali-metal adsorption. Moreover, a new peak emerges at 1.61 eV following alkali-metal adsorption, and some miscellaneous peaks appear at energies greater than 12.46 eV in the spectra of K-, Rb-, and Cs-adsorbed g-GaN. These results indicate that the alkali-metal-adsorbed g-GaN materials show a wide range of adjustment in their absorption spectra. Furthermore, the absorption coefficients for pristine and alkali-metal-adsorbed g-GaN are related to the imaginary part and the extinction index, as shown in Figs. 6b and 8c. As shown in Fig. 7b, the values for *n*(*0*) are 1.22 (pristine), 1.53 (Li), 1.78 (Na), 1.89 (K), 1.99 (Rb), and 1.99 (Cs). The *n*(*0*) values for pristine g-GaN and alkali-metal-adsorbed g-GaN are slightly lower than those obtained for pristine GaN nanowires and alkali-metal-adsorbed GaN nanowires [12]. With increasing photo energy, the refractive index of pristine g-GaN reaches a maximum value of approximately 1.65 at 5.88 eV, whereas the refractive indices of alkali-metal-adsorbed g-GaN reach a maximum value of approximately 1.75–2.25 at 0.7–2 eV. In addition, the refractive indices of pristine g-GaN and alkali-metal-adsorbed g-GaN reach a minimum value of approximately 11.41 eV. Finally, the refractive indices remain unchanged with a value of 0.91 when the photo energy is greater than 15 eV.

The reflectivity coefficient *R*(*ω*) for pristine and alkali-metal-adsorbed g-GaN is shown in Fig. 8a. A strong reflection peak is located at 11.3 eV for pristine g-GaN; however, the peak intensity decreases after alkali-metal adsorption. Moreover, a new reflection peak emerges in the low-energy region (0–2.5 eV), which indicates that the reflection spectrum is extended after alkali-metal adsorption. The energy-loss function *L*(*ω*) for pristine and alkali-metal-adsorbed g-GaN is shown in Fig. 8b; the data show that the most prominent peak for pristine g-GaN is located at approximately 11.57 eV, whereas the most prominent peak for alkali-metal-adsorbed g-GaN appears at 11.12 eV. The peak intensity for the alkali-metal-adsorbed g-GaN is lower than that for the pristine g-GaN; thus, the energy loss is slower for electron transmission in alkali-metal-adsorbed g-GaN. In addition, the alkali-metal-adsorbed g-GaN is a stable compound. The extinction coefficient *K*(*ω*) of pristine and alkali-metal-adsorbed g-GaN is shown in Fig. 8c. The extinction coefficient for the alkali-metal-adsorbed g-GaN is similar to the reflectivity coefficient. Thus, the optical properties of g-GaN can be tuned via the adsorption of alkali-metal atoms, which is useful for the fabrication of optoelectronic devices.

## Conclusions

The electronic and optical properties of alkali-metal-adsorbed g-GaN systems were investigated using density functional theory. The results are summarized as follows: (1) all the alkali-metal-adsorbed g-GaN are rather stable with the most stable adsorption site being the T_M_ site. (2) The adsorption of alkali metal atoms on g-GaN occurs via chemisorption. (3) An n-doping behavior can be found in g-GaN after the adsorption of alkali-metal adatoms. (4) The work function of g-GaN is considerably reduced following alkali-metal adsorption, with the Cs-adsorbed g-GaN system showing the minimum work function of only 0.84 eV, thus, the Cs adsorbed g-GaN system has potential application in field-emission devices. (5) Alkali-metal adsorption can lead to an increase in the static dielectric constant and extend the absorption spectrum of g-GaN. Consequently, the adsorption of alkali metals can be used to decorate and enlarge the optoelectronic properties of g-GaN, which can be used to produce photoelectric devices.

## Abbreviations

2D: Two-dimensional
GGA: Generalized gradient approximation
g-GaN: Graphene-like gallium nitride
PBE: Perdew-Burke-Ernzerhof
PDOS: Partial density of states
TDOS: Total density of states
